# A Diagnostic Dilemma: A Case of Complicated Pneumonia With Pyelonephritis and Subclinical Myocarditis

**DOI:** 10.7759/cureus.61853

**Published:** 2024-06-06

**Authors:** Kanishka Goswami, Gurjot Singh, Tanisha Sharma, Amna Farooq, Piyush Puri

**Affiliations:** 1 General Medicine, Maharaj Sawan Singh Charitable Hospital, Beas, IND; 2 Internal Medicine, Maharaj Sawan Singh Charitable Hospital, Beas, IND; 3 General Medicine, Maharishi Markandeshwar Medical College and Hospital, Solan, IND; 4 Medicine, Shaikh Khalifa Bin Zayed Al-Nahyan Medical and Dental College, Lahore, PAK; 5 Internal Medicine, Adesh Institute of Medical Science and Research, Bathinda, IND

**Keywords:** community-acquired pneumonia, complicated pneumonia with subclinical myocarditis, organizing pneumonia with pyelonephritis, atypical features, complicated pneumonia

## Abstract

A 41-year-old woman presented with a 3.5-month history of fever, weakness, productive cough, and burning micturition along with generalized weakness and significant weight loss. Chest X-ray revealed bilateral infiltrates and bilateral pleural effusion, and the workup suggested community-acquired pneumonia (CAP). However, the course was complicated by persistent fevers, elevated inflammatory markers, elevated N-terminal pro-B-type natriuretic peptide (NT-proBNP), and pelvic fluid collection.

Extensive investigations, including bronchoscopy and lung biopsy, failed to identify a specific pathogen. Pulmonary vasculitis and lymphoma were ruled out. Antibiotic and corticosteroid therapy resulted in clinical improvement. While the cause remains unknown, brucellosis and aspergillosis were considered but ruled out with advanced testing. The underlying etiology remains elusive, highlighting the diagnostic challenges in CAP with atypical presentations.

## Introduction

Community-acquired pneumonia (CAP) typically presents with a well-defined constellation of symptoms and responds favorably to antibiotics. However, a subset of patients deviates from this classic presentation, exhibiting a prolonged and complicated course of antibiotics and corticosteroids [1,2] that necessitates a more nuanced diagnostic approach [3,4]. This report describes the case of a 41-year-old woman with no significant past medical history who presented with a 3.5-month history of fever, generalized weakness, productive cough, burning micturition, and significant weight loss. Chest X-ray [5] revealed bilateral infiltrates, further suggesting pneumonia. However, the clinical picture was complicated by persistent fevers, elevated inflammatory markers [6], negative galactomannan test [7], and the presence of pleural effusions. These atypical features, combined with a negative initial workup, highlight the diagnostic challenges encountered in managing complex cases of complicated pneumonia or in a broader umbrella term organizing pneumonia [8-10] with unclear pathology.

## Case presentation

Case History

A 41-year-old woman with no significant past medical history presented with a 3.5-month history of fever (up to 101.5°F), generalized weakness, productive cough with yellow sputum, burning micturition, and significant weight loss. On examination, she was febrile (100°F) with bilateral chest crepitations. No history of asthma or skin lesions was reported. There was no family history of autoimmune disorders.

Investigations

**Figure 1:**
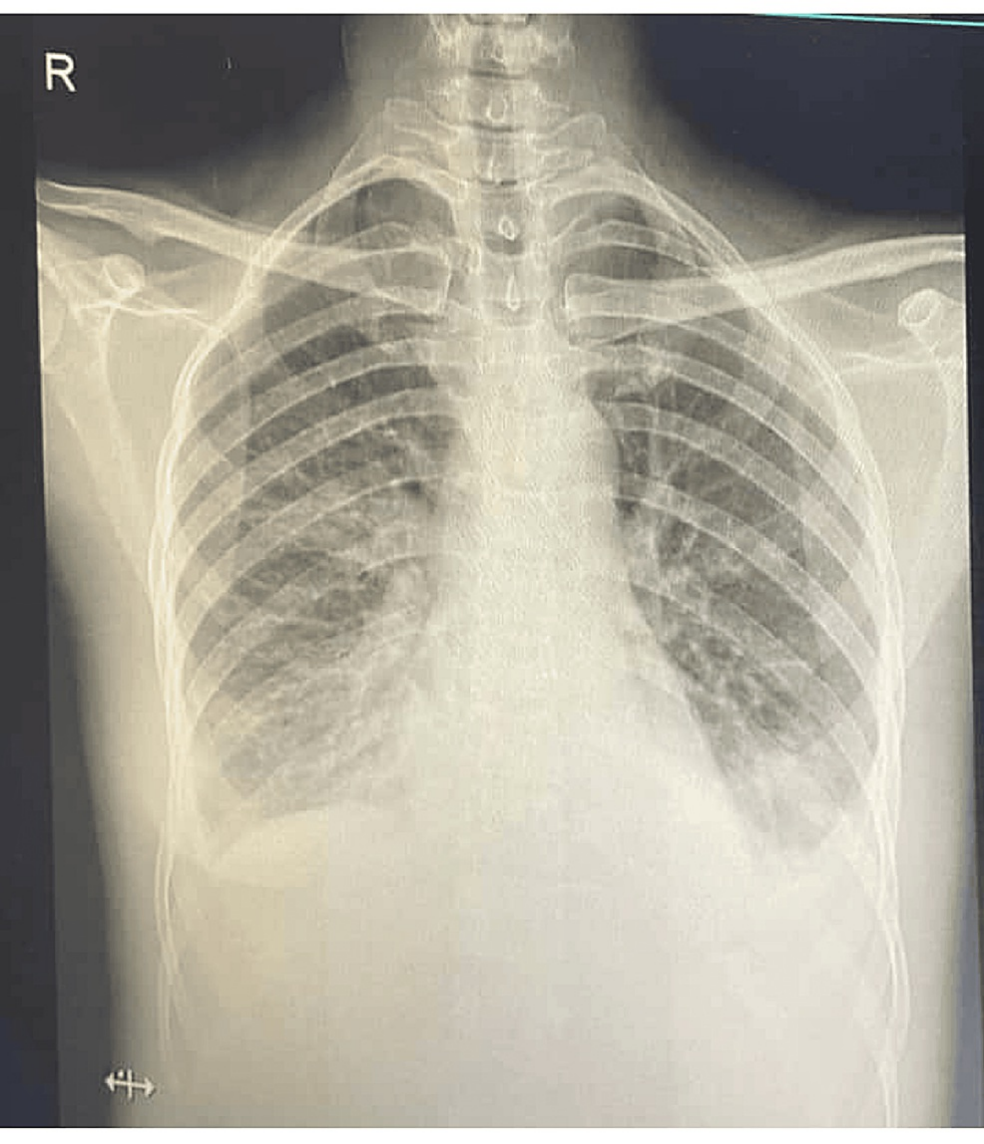
Chest X-ray

Chest X-ray revealed bilateral infiltrates and pleural effusions (Figure 1). Laboratory findings showed elevated inflammatory markers (CRP, ESR, ferritin), leukocytosis, and mild liver function test abnormalities. Urinalysis demonstrated pyuria. Sputum ZN Stain, IGRA yielded negative results. Sputum culture yielded *Acinetobacter baumannii*. Blood and urine cultures were negative. Viral serologies for common infections were negative. Autoimmune workup (anti-neutrophil cytoplasmic antibodies [ANCA], antinuclear antibody [ANA]) and galactomannan testing were negative. ECG was reported to have normal sinus rhythm (Tables 1-4).

**Table 1:** Hematological studies WBC, white blood cells; RBC, red blood cells; MCV, mean corpuscular volume; MCH, mean corpuscular hemoglobin; MCHC, mean corpuscular hemoglobin concentration; RDW, red cell distribution width; ESR, erythrocyte sedimentation rate.

Hematology	Results	Reference Range
Wbc count (1000/cumm)	13.1	12-15
Platelet count (1000/cumm)	549	150-450
RBC count (million/uL)	3.87	4.5-5.1
MCV (fL)	84.5	80-100
MCH (pg)	26.5	27.5-33.2
MCHC (g/dL)	31.4	33.4-35.5
RDW (%)	13.6	11.6-14.0
Neutrophils (%)	78	40-80
Lymphocytes (%)	14	20-40
Eosinophils (%)	04	1-6
Monocytes (%)	01	2-10
Basophils (%)	00	0-1
ESR (mm/h)	20	0-20

**Table 2:** Biochemical studies

Biochemistry Tests	Results	Reference Range
Urea (mg/dL)	10.7	15-40
Creatinine (mg/dL)	0.9	0.5-1.3
Total bilirubin (mg/dL)	0.5	0.2-1.0
Direct bilirubin (mg/dL)	0.1	<0.2
Indirect bilirubin (mg/dL)	0.4	0.2-0.8
Aspartate aminotransferase (U/L)	55.9	15-37
Alanine aminotransferase (U/L)	147.0	14-59
Alkaline phosphatase (U/L)	217.0	<98
Lactate dehydrogenase (U/L)	195.0	<250
Total protein (g/dL)	5.9	6.4-8.3
Albumin (g/dL)	2.3	3.2-4.5
Globulin (g/dL)	3.6	1.9-3.5
Gamma GT GGT (U/L)	85.0	<42

**Table 3:** Microbiological studies BAL, bronchoalveolar lavage.

Microbiology Tests	Results
Specimen: BAL fluid; Method: Gram stain	A moderate number of pus cells, occasional epithelial cells, and occasional Gram-positive cocci were seen. *Leuconostoc mesenteroides* ssp clindamycin-sensitive, teicoplanin-sensitive, penicillin-sensitive
Galactomannan test	Negative
Sputum ZN stain	No acid-fast bacilli seen
Sputum culture and sensitivity	Normal upper respiratory tract commensal flora grown after 48 h of aerobic incubation
Tropical fever detection by RT-PCR	Negative
Urine culture and sensitivity	No organisms grown after 48 h of aerobic incubation

**Table 4:** Miscellaneous studies NT-proBNP, N-terminal pro-B-type natriuretic peptide.

Miscellaneous	Results	Reference Range
NT-proBNP (ng/L)	293	70-133
D-dimer (mcg/L)	862	80-583
Procalcitonin-C (ng/mL)	0.14	<0.05 - healthy individuals; <0.5 - low risk of progression to severe systemic sepsis; 0.5-2.0 - moderate risk of progression to severe systemic sepsis; 2.00-10.00 - high risk of progression to severe systemic sepsis

Imaging

Chest CT scan showed patchy consolidation with ground-glass opacities in bilateral lungs and enlarged mediastinal lymph nodes. Chest ultrasound (USG) revealed blunted costophrenic angles. USG-guided fine-needle aspiration cytology (FNAC) of the left lower lung suggested an inflammatory lesion. Abdominal ultrasound showed mild hepatomegaly and a small liver cyst. Echocardiography revealed possible mitral valve prolapse (Table 5).

**Table 5:** Imaging studies R/M, routine and microscopy; LV, left ventricular; USG, ultrasound; FNAC, fine-needle aspiration cytology.

Imaging Studies	Results
CECT	• Large area of dense consolidation in the left lower lobe. • Multiple small patchy areas of consolidation scattered in both lung parenchyma. • Bilateral minimal pleural effusion with underlying passive atelectasis of lung parenchyma. • Few subcentimeter to centimeter pre/bilateral paratracheal, right hilar, and subcarinal lymph nodes, with the largest one measuring 1.5x1.4 cm. Imaging features are suggestive of infective etiology.
USG abdomen	Mildly hypoechoic left kidney - To correlate clinically to look for pyelonephritis. • Bulky uterus. • Few fine-moving internal echoes and debris were noted within urinary bladder - To correlate with urine R/M. Right-sided pleural effusion.
Echocardiography	Mild mitral valve prolapse was observed. The valve was not thickened and not calcified. The mitral valve score was 4/16. No mitral valve stenosis was observed. Estimated LV muscle mass (Deveruex) = 117.27 g or 71.07 g/m^2^ (normal: 43-95 g/m^2^). 2D ejection fraction: 60%.
Ultrasound of chest	Bilateral moderate pleural effusion
USG-guided FNAC left lower lung	Cytosmears examined show inflammatory cell infiltrate predominantly comprising neutrophils along with few lymphocytes, histiocytes, and few scattered cystic macrophages in the hemorrhagic background. A few small clusters of epithelial cells were seen showing reactive atypia. Overall cytomorphological features are suggestive of an inflammatory lesion.

Bronchoscopy and lung biopsy

Bronchoscopy with bronchoalveolar lavage showed neutrophilic inflammation with *Leuconostoc mesenteroides* on culture. Lung biopsy demonstrated neutrophilic inflammation but lacked evidence of malignancy or specific infectious agents on microscopy or special stains.

Management

The patient initially received empiric broad-spectrum antibiotics (linezolid, feropenem) based on culture results. Due to persistent fevers and inflammation, corticosteroids (prednisolone) were added. The patient showed a gradual improvement with resolution of fevers and improved oxygen saturation but relapse occurred as soon as prednisolone was tapered and discontinued, thus necessitating restarting of prednisolone and maintenance.

Consent

Written informed consent was obtained from the patient for publication of this case report.

## Discussion

This case highlights the challenges in diagnosing and managing complicated pneumonia with atypical features. Despite initial antibiotic therapy, the patient's course was complicated. Extensive investigations failed to identify a definitive pathogen, ruling out infective pathology as described by Ito and Ishida [5]. Tuberculosis [11] was also diligently ruled out with advanced diagnostic tests. Pulmonary vasculitis [3,4,6] and lymphoma [9] were excluded given mild leukocytosis and the clinical and pathological findings, as suggested by multiple studies including those of Huang et al. [3], Cherian et al. [4], and Addison et al. [6]. Brucellosis [8] and aspergillosis [4,7] were also considered but excluded with advanced testing, as suggested by He et al. [8] and Lim et al. [7]. A combination of antibiotics and corticosteroids led to clinical improvement, as suggested by Cendon et al. [1].

It is crucial to note that the patient's symptoms re-emerged upon tapering and eventual discontinuation of prednisolone. This phenomenon aligns with established knowledge that relapses are frequent in organizing pneumonia, particularly when treated with steroids [2]. These relapses often occur during the delicate phase of prednisolone dose reduction (typically around 5-10 mg) or within a few months of completely stopping the medication, as documented by Radzikowska and Fijolek [2]. This highlights the critical need for close follow-up examinations, especially during the first year after treatment completion.

## Conclusions

This case report underscores the significant challenge posed by diagnosing and managing complicated pneumonia with atypical features. The patient presented with a constellation of symptoms that defied easy categorization and resolution with initial antibiotic therapy. The extensive workup, encompassing blood tests, cultures, imaging studies, and even invasive procedures like bronchoscopy and lung biopsy, failed to pinpoint a specific pathogen.

This highlights the limitations of current diagnostic tools in such complex cases. While diagnoses like tuberculosis and pulmonary vasculitis along with other autoimmune pathologies, lymphoma, brucellosis, and aspergillosis along with other infective etiologies were entertained, they were ultimately ruled out based on clinical presentation, pathology, and advanced testing. The eventual improvement with a combination of antibiotics and corticosteroids suggests an underlying inflammatory process, but the exact etiology remains elusive.

This case serves as a valuable reminder for clinicians to consider atypical presentations of pneumonia and tailor their diagnostic and therapeutic strategies accordingly. A comprehensive approach that integrates clinical features, laboratory findings, and imaging studies is crucial for navigating such complex cases. By improving our understanding of atypical pneumonia presentations, we can strive for earlier diagnosis and more effective management strategies, ultimately leading to better patient outcomes.
